# Support for tobacco endgame approaches: Results from a web-based survey of stakeholders from 28 African countries

**DOI:** 10.18332/tid/210669

**Published:** 2025-11-07

**Authors:** Catherine O. Egbe, Mukhethwa Londani, Siphesihle Gwambe, Leonce Sessou, Omotayo F. Fagbule, Stella A. Bialous

**Affiliations:** 1 Mental health, Alcohol, Substance use and Tobacco Research Unit, South African Medical Research Council, Pretoria, South Africa; 2 School of Nursing and Public Health, Department of Public Health Medicine, Faculty of Health Sciences, University of KwaZulu-Natal, Durban, South Africa; 3 Department of Public Health, Faculty of Health Science, Sefako Makgatho Health Sciences University, Pretoria, South Africa; 4 Directorate of Research and Innovation, Tshwane University of Technology, Pretoria, South Africa; 5 Africa Tobacco Control Alliance, Lome, Togo; 6 Department of Periodontology and Community Dentistry, Faculty of Dentistry, College of Medicine, University of Ibadan, Ibadan, Nigeria; 7 School of Public Health, University of Nevada, Reno, Reno, United States; 8 School of Nursing, University of California San Francisco, San Francisco, United States; 9 Center for Tobacco Control Research and Education, University of California San Francisco, San Francisco, United States

**Keywords:** tobacco endgame, Africa stakeholders, tobacco control, strategies to end tobacco

## Abstract

**INTRODUCTION:**

The tobacco ‘endgame’ concept proposes moving beyond traditional tobacco control measures towards a tobacco-free future. The aim of this study is to investigate the perceptions of tobacco control stakeholders in Africa on their agreement with what endgame approaches are suited for the region to achieve a tobacco-free society.

**METHODS:**

Data were collected using a web-based cross-sectional survey hosted on Redcap. A total of 146 stakeholders from 28 African countries took the survey. Participants rated agreement with 11 proposed endgame approaches drawn from the literature and the qualitative phase of this study. Descriptive analysis was used to summarize stakeholders' level of agreement while bivariate (chi-squared and Fisher’s exact tests) and adjusted modified Poisson regression analyses examined association between agreement/disagreement to endgame approaches and demographic factors. Data were analyzed using STATA v17.

**RESULTS:**

All participants agreed to an integrated endgame approach while over 90% agreed with six measures (having non-addictive cigarettes, making cigarette unappealing, tobacco-free generation, regulated market model, quota/sinking lid and specific approaches for Africa). Agreements ranged from 70–85% for price caps, performance-based regulation, and non-combustible nicotine products, while only 35% supported government takeover of tobacco companies. Adjusted Poisson regression analyses showed that female stakeholders were less likely to support restricting tobacco sales by year of birth (relative risk ratio, RRR=0.89) and price caps (RRR=0.78), while PhD holders were more likely to support restricting tobacco sales by year of birth (RRR=1.29) and price caps (RRR=1.27). Stakeholders from Southern Africa were less likely to support a state takeover of tobacco companies (RRR=0.40) and performance-based regulation (RRR=0.76). Having more than 20 years of tobacco control experience lowered the support of price caps endgame measures (RRR=0.45).

**CONCLUSIONS:**

Policymakers are encouraged to use insights from this study to consider multifaceted approaches aimed at addressing the problem of commercial tobacco in the African region and pave the way for a tobacco-free Africa.

## INTRODUCTION

Tobacco use is a leading cause of preventable death worldwide, responsible for over 8 million deaths annually^1^. In Africa, the burden of tobacco use remains a significant public health concern, with a 2025 estimate of those smoking tobacco pegged at 84 million^2^. The tobacco epidemic in Africa is exacerbated by increasing levels of tobacco use initiation, particularly among the youth^3^, as well as the aggressive marketing strategies of the tobacco industry targeting this region^4^. Despite various tobacco control measures implemented across the continent, the rate of decline in tobacco use is slow^5,6^, necessitating more radical and comprehensive strategies to achieve a tobacco-free future. The prevalence of tobacco use in Africa is expected to increase due to increased marketing tactics by the tobacco industry^7,8^. Furthermore, it has been projected that tobacco use within the region would be higher than in LMICs by 2025^7,9^.

The tobacco endgame concept proposes moving beyond traditional tobacco control measures towards a tobacco-free future where commercial tobacco products are either phased out or significantly restricted in their use and availability^10^. While endgame strategies differ in method, policy approach, or specific measurable outcomes, one of the ultimate goals remains the same: the near or total elimination of commercial tobacco use in society^11,12^.

Endgame strategies aim to achieve a society free from tobacco use, often through innovative regulatory approaches that go beyond traditional tobacco control measures^11^. These strategies include policies such as regulating nicotine levels to make cigarettes non-addictive, restricting tobacco sales based on the year of birth (tobacco-free generation), redesigning cigarettes to make them unappealing, and implementing performance-based regulations that incentivize the reduction of tobacco use^11^.

In recent years, the concept of a tobacco endgame has gained momentum as countries and health organizations, particularly those in high-income countries, aim to drastically reduce tobacco use and eliminate tobacco-related harm^11-15^. Though the prevalence of tobacco use in African countries is relatively lower than in other regions, the inequitably high overall disease burden from tobacco use, the lack of significant reduction in tobacco use on the continent, and the fact that the tobacco industry is focused on expanding their market in Africa, provides further justification for the implementation of culturally specific endgame strategies on the continent.

To have effective endgame strategies, experts in tobacco control in Africa would need to be the vanguards of researching what endgame strategies would be effective in the continent, adapt these strategies to individual countries, and advocate for the adoption and implementation of these strategies by African governments. Similarly, according to Thomas et al.^14^, the development and implementation of effective endgame strategies often encounter significant political opposition, driven by intense industry lobbying. Hence, all stakeholders, particularly the experts in tobacco control, must have a good understanding of the different endgame strategies, be abreast of the ethical dilemmas, and anticipate and address potential implementation challenges.

Given the unique socio-economic and cultural contexts of African countries, it is crucial to understand the perspectives of professionals involved in tobacco control on the continent. These professionals play a pivotal role in shaping and implementing tobacco control policies, and their insights can inform the development of effective endgame strategies tailored to the African context. There is limited research on the perspectives of African tobacco control professionals regarding tobacco endgame strategies. This study seeks to address this gap by exploring their views on different endgame approaches, measuring their level of agreement, and identifying sociodemographic factors that influence their support for specific strategies.

## METHODS

### Research design

This study utilized a quantitative research design. This design allowed the researchers to investigate support for the implementation of various endgame strategies. A web-based cross-sectional survey was conducted for a period of 3 months from September to November 2023.

### Population

Participants in this study comprised individuals working in the tobacco control field and residing in one of the 48 countries in the Sub-Saharan African (SSA) region. These individuals include academics, advocates and government officials.

### Sample and sampling techniques

The sample of the study was derived from the African Tobacco Control Alliance (ATCA) database of 562 stakeholders in the WHO African Region. ATCA is a network of civil society organizations and non-governmental organizations with presence in 39 countries from the WHO African Region and they work to limit the detrimental impact of tobacco on the health and well-being of Africans^16^. All persons on the ATCA database were included in the study and were emailed links to the survey. The response rate was 26.0%.

### Measures

*Sociodemographic characteristics*

The sociodemographic data collected included: country of origin (categorized into the four sub-regions in SSA namely Western, Southern, Central, and Eastern Africa), sex (male, female), age (24–44 and ≥45 years), education level (diploma/other, Bachelor’s or equivalent degree, Master’s, and PhD or equivalent degree), years worked in tobacco control (<10, 10–19 and ≥20 years), current employer (government and non-government), current employment sector (advocacy, research/university, government ministry/department, other), and ever used tobacco (yes, no).

*Endgame approaches*

Participants were assessed for their level of agreement with 11 endgame approaches with sub-questions to capture aspects these approaches entail. These endgame approaches were curated from the literature as well as from findings from the qualitative phase of this study^17^. The 11 endgame approaches included: regulating nicotine levels, redesigning the cigarette to make it unappealing, restricting sales by year born, measures to emphasize the advantage of non-combustible nicotine products over combustible tobacco products, regulated market model, state takeover of tobacco companies, performance based regulation, quota or sinking lid approach, price caps on tobacco products, integrated approach and specific approach for Africa (see questionnaire in Supplementary file Material S1). An example of the question structure is shown below for the ‘Quota/ sinking lid’ endgame approach.

*Introduction statement: The following will result in the end of availability of smoked tobacco and near zero smoking prevalence:*

1. *Reducing smoked tobacco supply quotas for manufacturers and importers*
2. *Smoking cessation support, mass media campaigns and stronger marketing of tobacco cessation*
3. *Strict regulation of the retail of tobacco products*

*Response*

Participants were asked whether they agree or disagree with the questions under each endgame approach using a 4-point Likert scale. A combination of responses for each question under each approach formed the parent variable. The responses ‘agree’ or ‘disagree’ were created by combining the ‘strongly agree’ and ‘agree’ responses and the ‘strongly disagree’ and ‘disagree’ responses, respectively.

### Ethical considerations

Ethical clearance to conduct this study was obtained from the Human Research Ethics Committee of the South African Medical Research Council (Ref: EC014-4/2023). The link to the survey directed the participants to an information sheet/consent form in which participants were asked to indicate their intention to voluntarily participate in the study by digitally signing the informed consent form to proceed to the survey. Participants were also assured anonymity and confidentiality throughout the study. Publication of the information shared has been de-identified except for participants’ country name.

### Data collection instrument and procedure

The survey data were collected from September to November 2023, and managed using REDCap electronic data capture tools^18^ hosted by the South African Medical Research Council. REDCap is a web-based software developed to capture quantitative research data^19^.

The data collection instrument was a web-based structured questionnaire on 4-point Likert scale formatted responses. The questionnaire was available to participants in both English and French languages as they are the two major languages for communication in the SSA region. The survey link was sent to participants via email; participants were able to complete the survey anonymously. Daily monitoring of survey completion or attempts was done by the data manager. After the closure of the survey, data collected using the REDCap platform were exported to excel and cleaned before being exported to STATA version 17. All data collected were stored on password protected laptops of the data manager and principal investigator.

### Data analysis

Data were analyzed using STATA version 17. Descriptive analysis was conducted to determine frequencies and percentages to explore sample characteristics and the level of agreement with endgame approaches. Bivariate analyses were performed using chi-squared and Fisher’s exact tests to determine the association between agreement/disagreement with endgame approaches and participants’ region of origin, gender, age, education level, years worked in tobacco control and current employment. Adjusted modified Poisson regression analysis was performed to assess the factors associated with agreement to endgame approaches (such as regulate nicotine levels to make cigarettes non-addictive or less addictive; redesign the cigarette to make it unappealing; restrict sales by year born; and advantage of non-combustible nicotine products over combustible tobacco products). A p<0.05 was considered statistically significant for all analyses. All statistical tests conducted were two-tailed. A small proportion of participants had missing data, and were excluded on a case-by-case basis for each analysis.

## RESULTS

### Sample characteristics of the participants

Table 1 shows the distribution of sample characteristics among the participants. A total of 146 participants from 28 different African countries took part in the study. Of these participants, 47.3% were from West African countries, 61.4% were males, 61% were aged 24–44 years, 53.4% had a Master’s degree, 62.3% had <10 years of experience in tobacco control, and 75.3% worked for a non-government employer. More than half (56.2%) work in advocacy, and 86.3% said they have never used tobacco or nicotine. The number of participants per country ranged from 1 to 18.

**Table 1:** Sample characteristics of the participants, a cross-sectional web-based study, September to December 2023 (N=146)

*Characteristics*	*n*	*%*
**Number of countries**	28	
**Number of respondents per country** (range)	1–18	
**Sub-region of country of origin** (number of countries per region)		
Western Africa (12)	69	47.3
Southern Africa (8)	31	21.2
Central Africa (4)	14	9.6
Eastern Africa (4)	32	21.9
**Sex**		
Male	89	61.4
Female	56	38.6
**Age** (years)		
24–44	89	61.0
≥45	57	39.0
**Education level**		
Diploma/other	17	11.6
Bachelor’s or equivalent degree	30	20.6
Master’s	78	53.4
PhD or equivalent degree	21	14.4
**Years worked in tobacco control**		
<10	91	62.3
10–19	43	29.5
≥20	12	8.2
**Current employment**		
Government employee	25	17.1
Non-government employee	110	75.3
Other	11	7.5
**Sector in current employment**		
Advocacy	77	56.2
Research/university	21	15.3
Government ministry/department	14	10.2
Other	25	18.3
**Ever used any tobacco or nicotine product**		
Yes	20	13.7
No	126	86.3

### Stakeholders’ level of agreement with tobacco endgame approaches

**Figure 1:**
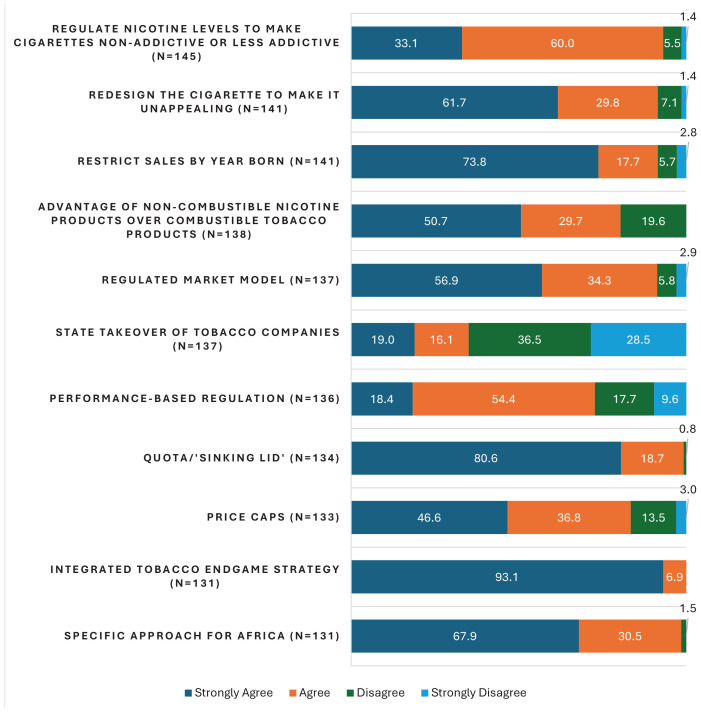
Participants’ level of agreement (%) with tobacco endgame approaches, a cross-sectional web-based study, September to December 2023 (N=146)

Figure 1 details participants’ level of agreement with various endgame approaches. For ease of interpretation, the variables which were measured on a 4-point Likert scale (strongly agree, agree, disagree, strongly disagree) were collapsed into two categories: agree (strongly agree and disagree) and disagree (strongly disagree and disagree). Participants supported regulating nicotine levels to make cigarettes non-addictive or less addictive (93.1%), redesigning the cigarette to make it unappealing (91.5%), restricting sales by year of birth (91.5%), advantage of non-combustible nicotine products over combustible tobacco products (80.4%), regulated market model (91.2%), performance-based regulation (72.8%), quota/sinking lid model (99.3%) and price caps (83.5%). The use of an integrated approach (100%) and designing a specific approach for Africa (98.5%) received the highest support while only 35% of participants supported State takeover of tobacco companies.

### Comparison of agreement to endgame approaches within demographic categories using chi-squared and Fisher’s exact tests

Table 2 shows that there was a significant difference in the support for various endgame approaches within subgroups of stakeholders. A higher proportion of stakeholders from Western Africa were in support of restricting sales by year born (p=0.010). A higher proportion of males were in support of redesigning the cigarette to make it unappealing (p=0.013) and having price caps (p=0.014). With regard to years of experience working in tobacco control, a higher proportion of those who have worked for 10–19 years support regulating nicotine levels to make cigarettes non/less addictive (p=0.034). Also, a higher proportion of those who have worked for <10 years support restricting sales by years born (p=0.047) and price caps (p=0.009). The sociodemographic variables did not differ by the following endgame approaches: ‘advantage of non-combustible nicotine products over combustible tobacco products’, ‘regulated market model’, ‘State takeover of tobacco companies’, and ‘performance-based regulation’.

**Table 2:** Agreement to endgame approaches by demographic characteristics, a cross-sectional web-based study, September to December 2023 (N=146)

*Variables*		*Regulate nicotine* *levels to make* *cigarettes non-addictive or less* *addictive* *n (%)*		*Redesign the* *cigarette to make* *it unappealing* *n (%)*		*Restrict sales by* *year born* *n (%)*		*Advantage of* *non-combustiblenicotine* *products over combustible* *tobacco products* *n (%)*		*Regulated market* *model n (%)*		*State takeover of* *stobacco companies* *n (%)*	*Performance-based regulation* *n (%)*	*Price caps*
**Sub-region of country of origin**														(0.835)
Western Africa		65 (95.59)		63 (95.45)		64 (96.97)		51 (78.46)		61 (93.85)		26 (40.00)	53 (82.81)	54 (84.38)
Southern Africa		28 (90.32)		26 (83.87)		29 (93.55)		26 (83.87)		26 (86.67)		5 (16.67)	20 (66.67)	26 (86.67)
Central Africa		12 (85.71)		13 (100.00)		9 (69.23)		11 (84.62)		12 (92.31)		6 (46.15)	8 (61.54)	9 (81.82)
Eastern Africa		30 (93.75)		27 (87.10)		27 (87.10)		23 (79.31)		26 (89.66)		11 (37.93)	18 (62.07)	22 (78.57)
p		0.356		0.149		**0.010**		0.931		0.648		0.113	0.097	(0.835)
**Sex**														
Male		83 (94.32)		81 (96.43)		79 (94.05)		66 (79.52)		76 (92.68)		29 (35.37)	60 (74.07)	71 (89.87)
Female		51 (91.07)		47 (83.93)		49 (87.50)		45 (83.33)		48 (88.89)		19 (35.19)	38 (70.37)	39 (73.58)
p		0.455		**0.013**		0.175		0.578		0.445		0.983	0.636	
**Age** (years)														(0.787)
24–44		84 (94.38)		79 (91.86)		79 (91.86)		70 (82.35)		79 (92.94)		26 (30.59)	60 (71.43)	69 (84.15)
≥45		51 (91.07)		50 (90.91)		50 (90.91)		41 (77.36)		46 (88.46)		22 (42.31)	39 (75.00)	42 (82.35)
p		0.444		0.843		0.843		0.472		0.368		0.163	0.649	0.787
**Education level**														
Diploma/other		16 (100)		15 (100)		12 (80.00)		12 (80.00)		14 (93.33)		5 (33.33)	11 (73.33)	13 (86.67)
Bachelor’s or equivalent degree		27 (90.00)		25 (89.29)		26 (92.86)		21 (75.00)		26 (92.86)		13 (46.43)	22 (78.57)	23 (82.14)
Master’s		73 (93.59)		70 (90.91)		70 (90.91)		61 (81.33)		67 (89.33)		23 (30.67)	52 (70.27)	57 (80.28)
PhD or equivalent degree		19 (90.48)		19 (90.48)		21 (100.0)		17 (85.00)		18 (94.74)		7 (36.84)	14 (73.68)	18 (94.74)
p		0.637		0.733		0.192		0.853		0.932		0.518	0.869	0.540
**Years worked in tobacco control**														
<10		85 (93.41)		81 (92.05)		84 (95.45)		70 (81.40)		79 (91.86)		27 (31.40)	66 (77.65)	74 (88.10)
10–19		41 (97.62)		39 (92.86)		36 (85.71)		33 (80.49)		38 (92.68)		18 (43.90)	25 (60.98)	32 (82.05)
≥20		9 (75.00)		9 (81.82)		9 (81.82)		8 (72.73)		8 (80.00)		3 (30.00)	8 (80.00)	5 (50.00)
p		**0.034**		0.428		**0.047**		0.751		0.360		0.371	0.141	**0.009**
**Current employment**														
Government employee	22 (88.00)		23 (95.83)		21 (87.50)		18 (78.26)		19 (86.36)		6 (27.27)		14 (66.67)	18 (85.71)
Non-government employee	103 (93.64)		97 (90.65)		99 (92.52)		84 (80.00)		97 (92.38)		37 (35.24)		76 (72.38)	84 (82.35)
Other	10 (100)		9 (90.00)		9 (90.00)		9 (90.00)		9 (90.00)		5 (50.00)		9 (90.00)	9 (90.00)
p	0.470		0.754		0.464		0.803		0.459		0.457		0.425	0.788

Chi-squared or Fisher’s exact tests were used depending on cell counts with Fisher’s used when expected frequencies (n) were <5. *p<0.05.

### Factors associated with agreement to endgame approaches

Table 3 presents the modified Poisson regression results examining the association between sociodemographic characteristics, years of experience, type of employment, and support for various tobacco endgame approaches. Female stakeholders were significantly less likely to support restricting tobacco sales by year of birth (RRR=0.89; 95% CI: 0.80–1.00, p=0.048) and price caps (RRR=0.78; 95% CI: 0.65–0.94, p=0.008) compared to male stakeholders. Stakeholders with a PhD (or equivalent degree) were significantly more likely to support restricting tobacco sales by year of birth (RRR=1.29; 95% CI: 1.01–1.65, p=0.040) and price caps (RRR=1.27; 95% CI: 1.04–1.55, p=0.019) compared with those holding a Diploma or other lower educational qualification. Stakeholders from Southern Africa were significantly less likely (compared to those from West Africa) to support a state takeover of tobacco companies (RRR=0.40; 95% CI: 0.17–0.93, p=0.033) and performance-based regulations (RRR=0.76, 95% CI: 0.57–1.00, p=0.049). Stakeholders with ≥20 years of experience in tobacco control were significantly less likely to support price caps compared to those with <10 years of experience (RRR=0.45; 95% CI: 0.26–0.77, p=0.003).

**Table 3:** Factors associated with agreement to endgame approaches, a cross-sectional web-based study, September to December 2023 (N=146)

*Variables*		*Regulate nicotine levels* *to make cigarettes* *non–addictive or less* *addictive*		*Redesign the cigarette* *to make it unappealing*		*Restrict sales by year* *born*		*Advantage of non–combustible nicotine* *products over* *combustible tobacco* *products*	
		*RRR (95% CI)*	*p*	*RRR (95% CI)*	*p*	*RRR (95% CI)*	*p*	*RRR (95% CI)*	*p*
**Sub-region of country of origin**									
Western Africa ®									
Southern Africa		0.97 (0.86–1.10)	0.654	0.90 (0.77–1.05)	0.183	0.97 (0.87–1.07)	0.530	1.11 (0.91–1.35)	0.289
Central Africa		0.88 (0.72–1.07)	0.199	1.05 (0.97–1.14)	0.216	0.73 (0.52–1.04)	0.080	1.10 (0.84–1.45)	0.488
Eastern Africa		0.97 (0.87–1.08)	0.581	0.92 (0.79–1.09)	0.347	0.94 (0.82–1.07)	0.356	1 (0.79–1.28)	0.987
**Sex**									
Male ®									
Female		0.95 (0.86–1.05)	0.322	0.88 (0.78–1.00)	0.057	0.89 (0.80–1.00)	**0.048**	1.02 (0.86–1.21)	0.797
**Age** (years)									
24–44 ®									
≥45		0.99 (0.91–1.09)	0.912	1.01 (0.89–1.14)	0.901	1.02 (0.90–1.16)	0.727	0.99 (0.80–1.22)	0.917
**Education level**									
Diploma/other ®									
Bachelor’s or equivalent degree		0.91 (0.80–1.03)	0.130	0.92 (0.81–1.05)	0.234	1.15 (0.90–1.47)	0.270	0.94 (0.67–1.32)	0.729
Master’s		0.95 (0.88–1.03)	0.204	0.95 (0.88–1.03)	0.203	1.16 (0.91–1.46)	0.225	1.02 (0.77–1.36)	0.870
PhD or equivalent degree		0.96 (0.84–1.10)	0.543	0.97 (0.80–1.17)	0.712	1.29 (1.01–1.65)	**0.040**	1.11 (0.79–1.57)	0.544
**Years worked in tobacco control**									
<10 ®									
10–19		1.06 (0.98–1.14)	0.158	0.98 (0.85–1.12)	0.771	0.89 (0.77–1.03)	0.121	0.99 (0.81–1.20)	0.916
≥20		0.79 (0.56–1.12)	0.181	0.87 (0.63–1.20)	0.391	0.78 (0.56–1.08)	0.132	0.84 (0.54–1.31)	0.442
**Current employment**									
Government employ ®									
Non-Government employee		1.07 (0.92–1.23)	0.387	0.96 (0.86–1.06)	0.395	1.10 (0.94–1.28)	0.251	1.02 (0.79–1.32)	0.875
Other		1.19 (0.99–1.42)	0.067	1.02 (0.83–1.25)	0.848	1.08 (0.85–1.38)	0.508	1.16 (0.84–1.61)	0.374
**Sub-region of country of origin**									
Western Africa ®									
Southern Africa		0.93 (0.80–1.07)	0.292	0.4 (0.17–0.93)	**0.033**	0.76 (0.57–1.00)	**0.049**	1.07 (0.89–1.29)	0.458
Central Africa		0.97 (0.82–1.15)	0.740	1.09 (0.54–2.21)	0.810	0.79 (0.50–1.24)	0.306	1.00 (0.74–1.34)	0.984
Eastern Africa		0.97 (0.83–1.14)	0.718	0.96 (0.53–1.73)	0.897	0.77 (0.57–1.05)	0.098	1.00 (0.82–1.23)	0.975
**Sex**									
Male ®									
Female		0.96 (0.85–1.08)	0.511	1.06 (0.67–1.67)	0.816	0.93 (0.75–1.17)	0.549	0.78 (0.65–0.94)	**0.008**
**Age** (years)									
24–44 ®									
≥45		0.70 (0.17–2.90)	0.621	2.07 (0.87–4.93)	0.102	1.62 (0.61–4.32)	0.331	1.56 (0.44–5.56)	0.491
**Education level**								
Diploma/other ®								
Bachelor’s or equivalent degree	1.01 (0.84–1.20)	0.952	1.38 (0.59–3.22)	0.463	1.05 (0.75–1.48)	0.775	0.91 (0.71–1.17)	0.468
Master’s	0.97 (0.82–1.15)	0.746	0.83 (0.38–1.85)	0.655	1.00 (0.72–1.39)	0.993	0.97 (0.79–1.18)	0.746
PhD or equivalent degree	1.06 (0.89–1.26)	0.491	1.18 (0.49–2.87)	0.714	1.00 (0.66–1.51)	0.990	1.27 (1.04–1.55)	**0.019**
**Years worked in tobacco control**								
<10 ®								
10–19	1.01 (0.88–1.16)	0.898	1.26 (0.74–2.15)	0.385	0.79 (0.59–1.04)	0.093	0.87 (0.72–1.06)	0.169
≥20	0.86 (0.64–1.17)	0.349	0.79 (0.31–2.04)	0.629	0.98 (0.66–1.44)	0.909	0.45 (0.26–0.77)	**0.003**
**Current employment**								
Government employ ®								
Non-government employee	1.07 (0.89–1.29)	0.470	1.34 (0.61–2.93)	0.469	1.10 (0.80–1.52)	0.554	1.02 (0.85–1.22)	0.809
Other	1.07 (0.82–1.39)	0.638	2.11 (0.82–5.42)	0.120	1.39 (0.94–2.05)	0.099	1.23 (0.92–1.65)	0.170

RRR: relative risk ratio. ® Reference categories. *p<0.05.

## DISCUSSION

This study captures the perspectives of tobacco control stakeholders in Africa on various endgame strategies, including regulating nicotine content to make cigarettes less attractive, restricting tobacco sales by birth year (tobacco-free generation), managing tobacco supply through government or non-profit agencies, implementing regulated market models, performance-based regulation, and gradually reducing the quota on tobacco products manufactured or imported (the ‘sinking lid’ approach).

While numerous studies on tobacco endgame strategies have been conducted in high-income countries, there is a significant gap in research focusing on Africa^11^. Most existing studies did not seek the views of tobacco control experts, who are pivotal in designing, developing, advocating, and monitoring the implementation of these strategies. This study aimed to fill that gap by providing insights from these key stakeholders.

The findings reveal strong support for regulating nicotine levels, redesigning cigarettes to make them unappealing, restricting sales by birth year, and regulated market model. This agreement aligns with global trends that emphasize innovative and aggressive measures to eliminate tobacco smoking^11^. Notably, almost all participating stakeholders strongly supported an integrated endgame strategy tailored specifically for Africa.

Developing Africa-specific tobacco endgame strategies is crucial due to the continent’s unique social, economic, cultural, and political contexts. African countries have diverse sociocultural landscapes where traditional beliefs and practices significantly influence tobacco use, necessitating culturally relevant public health messaging^20,21^. Economic factors, such as lower average incomes and the prevalence of informal markets^22^, require customized fiscal measures and considerations of economic dependence on tobacco farming^23^. Additionally, healthcare systems’ varying capacities to support smoking cessation and tobacco control interventions^24^ call for innovative approaches to overcome geographical and financial barriers to access cessation services. Governance and legislative frameworks differ across the continent, highlighting the need for strategies that can navigate and bolster these systems effectively^25,26^.

Moreover, the tobacco industry’s aggressive marketing and lobbying tactics in African countries necessitate strategies that can counter these efforts and address the industry’s economic influence^15^. The dual burden of infectious and non-communicable diseases complicates the public health landscape, requiring integrated tobacco control strategies that address tobacco-related diseases within limited healthcare financing. Community engagement and advocacy are also essential to educate the public about the harmful impact of commercial tobacco, as grassroots movements and public awareness campaigns must resonate with local populations and foster ownership of tobacco control measures. By addressing these specific challenges, tailored tobacco endgame strategies for Africa can more effectively reduce tobacco use and enhance public health outcomes across the continent.

Contrasting with the strong support for integrated endgame strategies, most participants opposed the idea of the state or government taking over of tobacco companies. This opposition likely reflects concerns about government’s capacity, potential conflicts of interest, or the feasibility of such interventions in African contexts. Notably, tobacco control stakeholders from Southern Africa were less supportive of government takeover compared to their Western African counterparts. This trend also extended to performance-based regulation, with Southern African experts showing less support than those from Western Africa.

In this study, sociodemographic characteristics significantly associated with less support for various endgame strategies included being female (tobacco-free generation and price caps), being from Southern Africa (State takeover and performance-based model) and having ≥20 years’ experience working in tobacco control (price caps). While those having a PhD were significantly associated with more support for tobacco-free generation and price caps.

A systematic review investigating public support for tobacco endgame strategies found that sociodemographic factors such as age, education level, and smoking status were significantly associated with support for various endgame policies^27^. These variations in level of support based on demographic characteristics suggest the need for tailored advocacy and educational efforts.

Despite these differences, the strong support for an integrated and Africa-specific approach underscores the importance of regional collaborations in developing and implementing endgame strategies. The unanimous backing for an integrated strategy highlights the necessity of adopting multifaceted and multisectoral approaches that address both tobacco supply and demand factors^28,29^. Policymakers and advocates should leverage existing regional bodies and frameworks to harmonize efforts and share best practices to further tobacco control in SSA.

### Limitations

This study’s limitations include the fact that it is limited to stakeholders who are in the ATCA database, and it uses a quantitative approach, which may have restricted the depth of information gathered from participants. While this study builds on a previous qualitative study, the aspects of tobacco endgame investigated in this study is slightly different from what was explored in the qualitative phase. Future research should adopt a qualitative approach to gain deeper insights into reasons behind the support or opposition to specific endgame approaches. Also, involving a larger number of the public (including those who smoke and those who do not smoke) would help to better measure public support for or against the implementation of endgame strategies in the African region. The small sample size limits the generalization of the results. The fact that very small numbers per country responded to the survey did not allow us to do country level analysis of stakeholders’ support for endgame policies. Also, no causal link can be drawn between working in tobacco control or any other sociodemographic characteristic and support/disagreement with these tobacco endgame policies. While participants’ tobacco control advocacy experience and demographic characteristics may introduce bias, this study’s key strength is being the first to examine public support for tobacco endgame strategies in Africa.

## CONCLUSIONS

Our study highlights strong support among African tobacco control stakeholders for comprehensive and innovative endgame strategies, with some variations by demographic characteristics. These findings provide a robust foundation for future research on tobacco endgame in the region and for policymakers and advocates to develop targeted interventions that align with the diverse needs and perspectives within the continent. As Africa continues to grapple with the tobacco epidemic, leveraging these insights can facilitate the effective implementation of endgame strategies and move closer to a tobacco-free future.

## Supplementary Material



## Data Availability

The data supporting this research are available from the authors on reasonable request. Requests will be considered on a case-by-case basis.
