# Supplementary material for: Support for tobacco endgame approaches: Results from a web-based survey of stakeholders from 28 African countries
Source: Tob Induc Dis. 2025 Nov 7;23:10.18332/tid/210669. doi: 10.18332/tid/210669 (PMC12598468; doi:10.18332/tid/210669)
Supplement: Supplementary file 1 [file TID-23-175-s1.pdf]

Tobacco Endgame in Africa Questionnaire: English version

Please complete the survey below.

Thank you!

This project is a collaboration between the South African Medical Research Council (SAMRC) and the Africa Tobacco Control Alliance (ATCA)

PARTICIPANT SHEET AND CONSENT FORM: PHASE 2 - SURVEY

RESEARCH TITLE: EXPLORING AN AFRICAN PERSPECTIVE TO A TOBACCO ENDGAME

Principle Investigator: Dr Catherine Egbe

Introduction

Greetings! This survey is being conducted by researchers from the South African Medical Research Council (SAMRC). We kindly request your participation in our study titled, Exploring an African perspective to Tobacco Endgame.

About the study

This study is for those who are 18 years and above residing in any of the countries in the Sub-Saharan part of Africa and who work in the field of tobacco control.

What we ask of you

The survey will take 10 to 15mins to complete. The survey is available in English or French languages and is anonymous. No personal details of yours is required.

Harms or discomfort

You may feel uncomfortable when answering the questions because of the sensitive information asked (especially if you are a current smoker or have smoked before). However, we assure you that all information provided by you will remain confidential.

Benefit for participating in the study

There are no direct benefits for participating this study. All the information collected from you will be used to assist for research purpose and to inform policy.

Participating in this study is voluntary

Participating in this study is strictly voluntary. You have the right to stop participating in the study at any given time without giving a reason.

Compensating

Please take note that you will not be compensated for this study.

Confidentiality

Your identity will remain confidential, during and after the study. All information you share with us will be used for research purpose only. All information and materials related to this study will be kept safe in a passworded laptop computer and after 15 years, the information will be destroyed.

People to contact about the study

This study has been approved by the SAMRC Human Research Ethics Committee and will be conducted accordingly in line with the International Declaration of Helsinki, and the South African Guidelines for Good Clinical Practice.

If you have any questions about your rights as a participant, any concerns or complains, you are urged to call the chairperson of the SAMRC Human Research Ethics committee researcher. Danie du Toit, at (021) 938 0687 or email adri.labuschagne@mrc.ac.za. If you have questions about the study you may call Dr Catherine Egbe on (012) 339 8504 from 8h00 to 16h30, or send an email to catherine.egbe@mrc.ac.za

Participant declaration

By continuing with this survey, I agree to participate in this study.
I declare that:

I have read through the details of the consent form and it is written in a language I am comfortable with. I understand that participating in the study is voluntary and there will be no bad outcomes if I choose not to participate in this study I am not forced to participate in this study I understand that I will get a copy of this form

Please check your desired option below.	◯ I agree to participate in this study<br>◯ I do not agree to participate in this study
Socio-demographic questions	
What is your country of origin?	________________________
What is your country of residence?	________________________
What gender do you identify as?	◯ Male<br>◯ Female<br>◯ Prefer not to say
How old are you (age in years)?	________________________
What is your highest level of education?	◯ No formal education<br>◯ Primary school education<br>◯ High school education<br>◯ Diploma<br>◯ Bachelor's Degree (or equivalent degree)<br>◯ Master's Degree<br>◯ PhD (or equivalent degree)<br>◯ Other
Please specify	________________________
How long have you worked in tobacco control? (in years)	________________________
What is your current employment status?	◯ Government employee<br>◯ Non-Governmental/Non-Profit Organization (NGO/NPO) employee<br>◯ Private company/organization employee<br>◯ Self-employed<br>◯ Unemployed

In what sector is your current employment?	◯ Advocacy<br>◯ Research/university<br>◯ Government ministry/department<br>◯ None of the above
Have you ever used any tobacco or nicotine product?	◯ Yes<br>◯ No
Do you currently use any tobacco or nicotine product (Example: cigarettes, waterpipe, snuff, oral nicotine pouch, electronic cigarettes)?	◯ Yes<br>◯ No
What type of tobacco or electronic cigarettes product do you currently use?	__________________________________
For how long have you been using this tobacco or nicotine product (please answer in years)?	__________________________________

Support for Tobacco Endgame Proposals

Each of the statements fall under one of the tobacco endgame strategies proposed by experts and summarized by McDaniel et al. (2015). Please read each statement carefully and indicate your agreement/disagreement with it.

Please tick on the right, the best option which fits with your agreements or disagreement with the statements on the left.

SA - Strongly Agree

A - Agree

D - Disagree

SD - Strongly Disagree

Regulate nicotine levels to make cigarettes non-addictive or less addictive

Intro statement: The following may lead to a reduction in nicotine addiction and protect health

	Strongly Agree	Agree	Disagree	Strongly Disagree
Regulating the marketing, advertisement and use of all electronic delivering systems	○	○	○	○
Making tobacco and nicotine products (like cigarettes and e-cigarettes) less attractive	○	○	○	○
Producing improved nicotine products which are not contaminated with metals or other substances from burning	○	○	○	○
Non-combustible nicotine products which are freely available as a substitute for combustible tobacco products	○	○	○	○
Regulating the content of nicotine in all tobacco products	○	○	○	○

Redesign the cigarette to make it unappealing

Intro statement: The following may lead to a reduction in cigarette smoking or nicotine addiction and protect health

	Strongly Agree	Agree	Disagree	Strongly Disagree
Removing all additives in cigarettes (like flavors)	○	○	○	○
Making smokers to obtain a yearly license in order to buy cigarettes	○	○	○	○
Having a limitation on the number of cigarettes a smoker can purchase daily or weekly	○	○	○	○
Making all new smokers take and pass a test of knowledge of the risk of smoking	○	○	○	○

Restrict sales by year born

	Strongly Agree	Agree	Disagree	Strongly Disagree
Individuals born in or after a specific year (agreed by government in consultation with the public) should be prohibited from buying tobacco products in order to reduce tobacco deaths	◯	◯	◯	◯

Advantage of non-combustible nicotine products over combustible tobacco products

Intro statement: The following could lead to the end of the tobacco epidemic

	Strongly Agree	Agree	Disagree	Strongly Disagree
Regulation of smokeless tobacco products (like snuff) in order for their use by smokers to be encouraged (e.g., marketing limited to current smokers, removal of additives)	○	○	○	○
Regulation of e-cigarettes in order for their use by smokers to be encouraged (e.g., marketing limited to current smokers, removal of flavors)	○	○	○	○
Increasing taxes on cigarettes and other combustible tobacco products	○	○	○	○

Regulated market model

Intro statement: The following will lead to the reduction of tobacco deaths

	Strongly Agree	Agree	Disagree	Strongly Disagree
Regulating the tobacco industry to encourage development of less harmful products	○	○	○	○
Regulating the tobacco industry to control their commercial communication through marketing	○	○	○	○

State takeover of tobacco companies

	Strongly Agree	Agree	Disagree	Strongly Disagree
If governments take over the manufacturing and supply of cigarettes or hand this to appointed non-profit companies, tobacco use can be phased out or reduced.	◯	◯	◯	◯

Performance-based regulation

Intro statement: Tobacco use and deaths caused by tobacco use can be reduced...

	Strongly Agree	Agree	Disagree	Strongly Disagree
If tobacco companies are allowed to determine how to meet goals for reducing smoking prevalence	○	○	○	○
If government should penalize tobacco companies if they fail to achieve a reduction in smoking prevalence within a reasonable time.	○	○	○	○

Quota/'sinking lid'

Intro statement: The following will result in the end of availability of smoked tobacco and near zero smoking prevalence

	Strongly Agree	Agree	Disagree	Strongly Disagree
Reducing smoked tobacco supply quotas for manufacturers and importers	◯	◯	◯	◯
Smoking cessation support, mass media campaigns and stronger marketing of tobacco cessation	◯	◯	◯	◯
Strict regulation of the retail of tobacco products	◯	◯	◯	◯

Price Caps

Intro statement: The following will reduce tobacco use and tobacco-related deaths

	Strongly Agree	Agree	Disagree	Strongly Disagree
Establishing an independent regulatory agency to set minimum and maximum wholesale prices (not retail price) for tobacco products	○	○	○	○
Establishing an independent regulatory agency to set minimum and maximum retail price for tobacco products	○	○	○	○

Integrated Tobacco Endgame Strategy

Intro statement: The following will reduce tobacco use and tobacco-related deaths

	Strongly Agree	Agree	Disagree	Strongly Disagree
By fully and quickly implementing the Framework Convention on Tobacco Control (international treaty on how to reduce tobacco use)	○	○	○	○
A gradual phasing out of the sale of tobacco products globally with an aim for a complete phase out in 2040	○	○	○	○
Strengthening tested approaches like improved health warning labels and graphic health warnings on tobacco product packages	○	○	○	○
Prohibiting tobacco industry contact with youth	○	○	○	○

Specific approach for Africa

Intro statement: Tobacco endgame can be achieved in Africa if:

	Strongly Agree	Agree	Disagree	Strongly Disagree
More people are educated about the importance of ending tobacco use and the tobacco epidemic	◯	◯	◯	◯
The manufacturing and sale of tobacco products is prohibited	◯	◯	◯	◯
Each country devises their own strategy to end tobacco use	◯	◯	◯	◯
Only after countries reach a smoking prevalence of less than 10%	◯	◯	◯	◯
More young people are encouraged to take up sports	◯	◯	◯	◯
Tobacco cessation services are provided in government hospitals to help more smokers quit	◯	◯	◯	◯
Tobacco farmers are assisted to stop farming tobacco	◯	◯	◯	◯
Herbal remedies are used to treat nicotine addiction	◯	◯	◯	◯

© 2025 Egbe C.O. et al.
